# DeepRSMA: a cross-fusion-based deep learning method for RNA–small molecule binding affinity prediction

**DOI:** 10.1093/bioinformatics/btae678

**Published:** 2024-11-14

**Authors:** Zhijian Huang, Yucheng Wang, Song Chen, Yaw Sing Tan, Lei Deng, Min Wu

**Affiliations:** School of Computer Science and Engineering, Central South University, Changsha 410083, China; Machine Intellection Department, Institute for Infocomm Research, Agency for Science, Technology and Research (A*STAR), Singapore 138632, Singapore; School of Computer Science and Engineering, Central South University, Changsha 410083, China; Bioinformatics Institute, Agency for Science, Technology and Research (A*STAR), Singapore 138671, Singapore; School of Computer Science and Engineering, Central South University, Changsha 410083, China; Machine Intellection Department, Institute for Infocomm Research, Agency for Science, Technology and Research (A*STAR), Singapore 138632, Singapore

## Abstract

**Motivation:**

RNA is implicated in numerous aberrant cellular functions and disease progressions, highlighting the crucial importance of RNA-targeted drugs. To accelerate the discovery of such drugs, it is essential to develop an effective computational method for predicting RNA–small molecule affinity (RSMA). Recently, deep learning-based computational methods have been promising due to their powerful nonlinear modeling ability. However, the leveraging of advanced deep learning methods to mine the diverse information of RNAs, small molecules, and their interaction still remains a great challenge.

**Results:**

In this study, we present DeepRSMA, an innovative cross-attention-based deep learning method for RSMA prediction. To effectively capture fine-grained features from RNA and small molecules, we developed nucleotide-level and atomic-level feature extraction modules for RNA and small molecules, respectively. Additionally, we incorporated both sequence and graph views into these modules to capture features from multiple perspectives. Moreover, a transformer-based cross-fusion module is introduced to learn the general patterns of interactions between RNAs and small molecules. To achieve effective RSMA prediction, we integrated the RNA and small molecule representations from the feature extraction and cross-fusion modules. Our results show that DeepRSMA outperforms baseline methods in multiple test settings. The interpretability analysis and the case study on spinal muscular atrophy demonstrate that DeepRSMA has the potential to guide RNA-targeted drug design.

**Availability and implementation:**

The codes and data are publicly available at https://github.com/Hhhzj-7/DeepRSMA.

## 1 Introduction

RNA is essential to the execution of various biological functions such as transcription and translation (Caprara and Nilsen 2000). The disruption of structure-related regulatory activities in many RNAs can result in various diseases (Costales *et al.* 2020), emphasizing their potential as drug targets. Due to the upstream position of RNA in the translation pathway, it is considered a desirable target for disease treatment. For example, inhibiting mRNAs with drugs can prevent the expression of downstream genes. Specifically, risdiplam is the first FDA approved small-molecule drug that directly targets human RNA to treat spinal muscular atrophy (SMA) (O’Keefe 2020). Risdiplam binds to the precursor messenger RNA (pre-mRNA) of the surviving motor neuron 2 (SMN2), consequently augmenting the synthesis of SMN proteins, which are pivotal for motor neuron functionality (Ratni *et al.* 2018). Similar to proteins, when an RNA exhibits pockets for binding to small molecules, it has the potential to act as a drug target (Warner *et al.* 2018). Therefore, in order to accelerate the development of RNA-targeted drugs, it is essential to predict the binding affinity between RNAs and small molecules (Childs-Disney *et al.* 2022).

Experimental affinity measurement methods provide valuable bioactivity data for small-molecule ligands. However, such methods face several challenges, including high costs and significant time requirements (Kairys *et al.* 2019). Computational methods can complement experimental methods by reducing the number of compounds to be tested. Previous computation methods can be divided into two categories. The first strategy is to directly evaluate the affinity between RNA and small molecule based on their features through a scoring function. For example, Guilbert and James (2008) docked the ligands by minimizing the root-mean-square-deviation-driven energy. Feng and Huang (2020) proposed ITScore-NL, which evaluates nucleic acid–ligand interactions by stacking interactions and electrostatic potentials. The second strategy is to use machine learning algorithms to extract features from RNA and small molecule for binding affinity prediction. For example, Grimberg *et al.* (2022) used deep convolutional networks, linear regression, and classification trees to predict small molecules binding to ribosomal hairpin 91. Szulc *et al.* (2023) developed a software FingeRNAt, which can encode several noncovalent interactions of RNA and ligand as fingerprints and combine with machine learning method to train a separate model for each molecular target to predict the RNA–small molecule interaction. Krishnan *et al.* (2024) proposed RSAPred that categorizes RNA into six subtypes and performs feature selection for each subtype. It then trains multiple affinity prediction models with distinct features for different RNA subtypes.

While previous methods have made significant strides in RNA–small molecule affinity prediction, there still exists ample room for further development. First, most of these methods focus on predicting the affinity for a specific RNA (Grimberg *et al.* 2022) or a specific RNA subtype (Krishnan *et al.* 2024), but they are not generalized for other RNAs. Second, various data modalities can help a model learn features from different perspectives, while existing methods typically consider either the graph or sequence modality of RNA and small molecule, preventing the model from capturing comprehensive features. Third, current methods ignore fine-grained information such as nucleotides of RNA and atoms of small molecules, restricting them to mine detailed features. Lastly, the interactions between specific nucleotides of RNA and atoms of small molecule are essential (Yu *et al.* 2020), while existing methods fail to consider such information for predicting RSMA.

To improve the accuracy of RNA–small molecule affinity prediction, we propose DeepRSMA, a novel cross-attention-based deep learning method for accurately predicting the binding affinity of RNA small-molecule ligands. Firstly, we design fine-grained feature extraction modules for RNA and small molecules, considering the nucleotide-level information for RNA and atom-level information for small molecule. Meanwhile, these modules process both features from sequence and graph perspectives to obtain sequence embedding and graph embedding, allowing the model to comprehensively mine the information contained in each pair of molecules. In order to empower DeepRSMA to recognize key regions of RNA–small molecule interactions, we design a cross-fusion module that can calculate the interaction between RNA nucleotides and small molecule atoms across sequence and graph perspectives, thereby obtaining cross embeddings. Finally, a prediction module is designed to combine the embeddings from feature extraction and cross-fusion modules for effective RNA–small molecule binding affinity prediction. Extensive test results under different settings and datasets show that DeepRSMA achieves the state-of-the-art performance. Meanwhile, the interpretability experiment and case study demonstrate the effectiveness and robustness of DeepRSMA.

## 2 Methods

### 2.1 Overview

**Figure 1.**
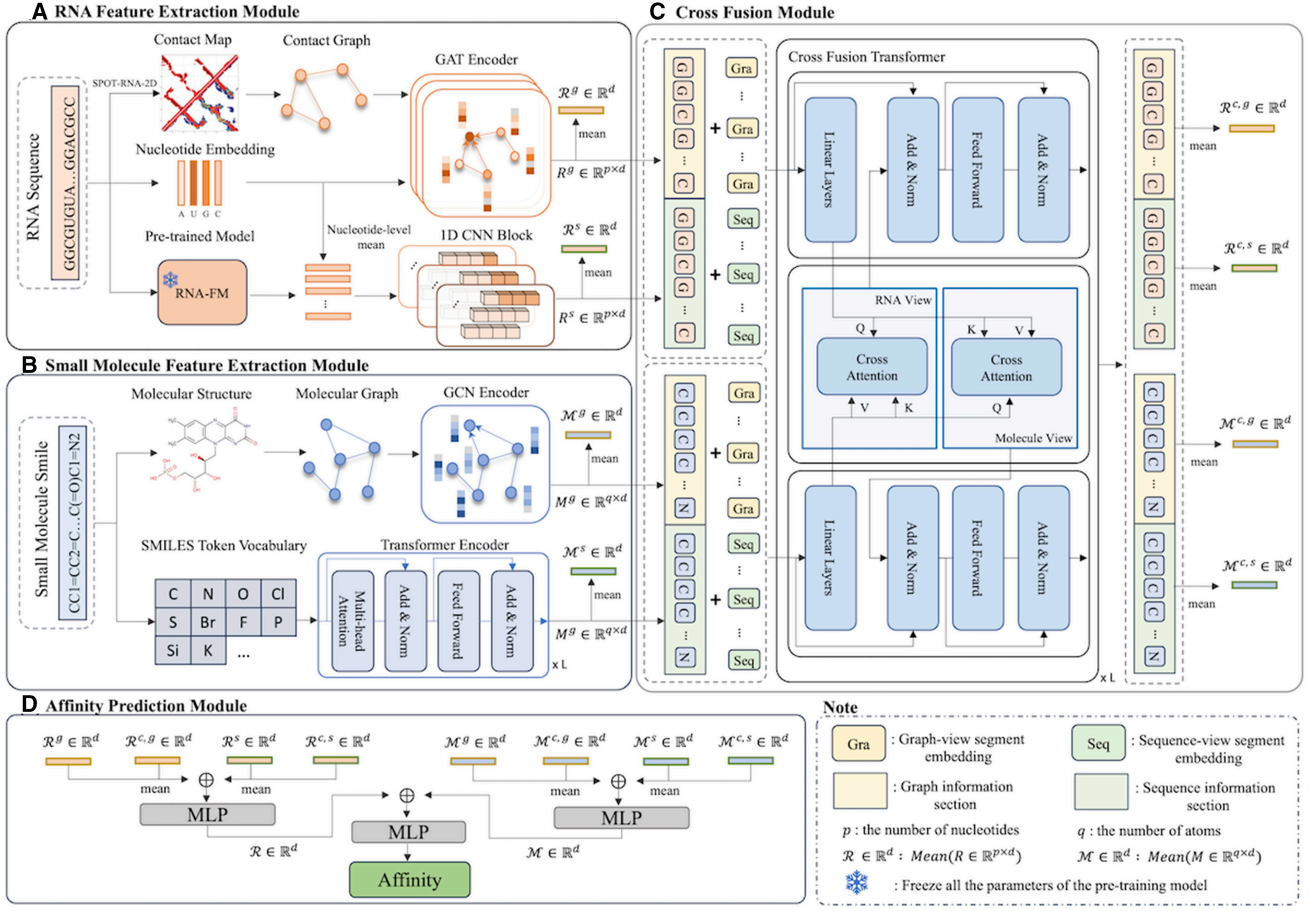
Overview of the DeepRSMA framework. (A, B) Feature extraction modules for RNA and small molecule, separately. (C) Cross-fusion module to integrate RNA and small molecule information from different views. (D) Affinity prediction module to combine the RNA and small molecule representations from (A), (B), and (C) and predict RNA–small molecule binding affinity values.

As illustrated in Fig. 1, our DeepRSMA comprises four modules: an RNA feature extraction module, a small molecule feature extraction module, a cross-fusion module, and an affinity prediction module. To leverage the features from different modalities, the RNA feature extraction module processes the RNA sequence into a contact map, nucleotide embedding, and pretrained embedding by RNA-FM (Chen *et al.* 2022). To obtain the RNA graph information, we use a graph attention network (GAT) (Veličković *et al.* 2017) encoder to combine contact map and nucleotide embedding. Meanwhile, the 1D convolutional neural network (CNN) (Krizhevsky *et al.* 2017) blocks are introduced to mine multiscale sequence information from nucleotide embedding and pretrained embedding. In the small molecule feature extraction module, we process SMILES into 2D molecular structure and SMILES token embedding. The graph convolutional network (GCN) (Kipf and Welling 2017) is used to extract the 2D structure information from the molecular graph. According to the transformer encoder, we can generate the small molecule embedding from SMILES sequence. To consider cross-information and the interaction of RNA and small molecule, we design a cross-fusion module that integrates RNA and small molecule information through a cross-fusion based transformer. Finally, the affinity prediction module predicts RNA–small molecule binding affinity by fusing the information from the RNA and small molecule.

### 2.2 RNA feature extraction module

#### 2.2.1 RNA graph information

To construct an RNA graph, we first employ SPOT-RNA-2D (Singh *et al.* 2022) to obtain distance-based contact maps for RNAs, which defines two nucleotides as being in contact if the distance of their nearest-heavy atoms is less than 8 Å. Specifically, we use an embedding layer to project each nucleotide into a dense continuous space to represent nodes of RNA graph. To capture the complex node relationships in the RNA graph, we then utilize GAT, a multihead attention-based method for learning node embedding. Taking nucleotide *i* in an RNA as an example, the attention score $\alpha_{in}$ between *i* and its neighbor node *n* is calculated as

(1) $$\alpha_{in}^{l}=\frac{\;\text{exp}(\text{LeakyReLU}(a^{T}[W_{d}r_{i}^{l},W_{d}r_{n}^{l}]))}{\sum_{m\in N(i)}\;\text{exp}\;(\text{LeakyReLU}(a^{T}[W_{d}r_{i}^{l},W_{d}r_{m}^{l}]))},$$

where LeakyReLU is an activate function, $r_{i}^{l}$ is the hidden representation of nucleotide *i* in the *l*th layer, *W_d_* is a trainable parameter, *a* is a learnable weight matrix, and N(i) is the neighbor nodes of *i*. After acquiring the attention coefficient, the representation of nucleotide *i* is calculated by a linear attention aggregation layer:

(2) $$r_{i}^{g,l}=\text{ReLU}\left(\alpha_{ii}^{l-1}W_{a}r_{i}^{g,l-1}+\sum_{n\in N(i)}\alpha_{in}^{l-1}W_{a}r_{n}^{g,l-1}\right),$$

where *W_a_* is a trainable parameter. Assuming an RNA has *p* nucleotides, the graph embedding for all the nucleotides is $R^{g}\in R^{p\times d}=[r_{1}^{g},r_{2}^{g},\ldots ,r_{p}^{g}]$. The RNA graph embedding is denoted as $R^{g}\in R^{d}=\text{Mean}(R^{g})=\frac{1}{p}\sum_{i=1}^{p}r_{i}^{g}$, where *d* is the dimension of hidden size.

#### 2.2.2 RNA sequence information

To capture RNA sequence features at different scales, we employ 1D CNN with convolution kernels of different sizes. To better capture RNA semantic information, 1D CNN block takes two different embeddings as inputs. The first embedding is an initial nucleotide embedding converted from one-hot encoding by using an embedding layer. Meanwhile, to incorporate RNA background knowledge into the model, we leverage the power of RNA-FM, an RNA foundation model pretrained on 23 million noncoding RNA sequences, for extracting the second embedding. Then, we average the nucleotide embedding and the pretrained embedding from RNA-FM at nucleotide level as the input. We pad the input with all zero vectors before the convolution operation to prevent the convolution operation from changing the length of the output embedding. Here, we define the input as I:[1,l]→R and the kernel function K:[1,k]→R. The convolution output *C*(*j*) between *I* and *K* is calculated as follows:

(3) $$C(j)=\sum_{i=1}^{k}I(j-i+k)\cdot K(i),$$

where *l* is the length of input RNA and *k* is the kernel size. Inspired by Wang *et al.* (2023), we employ three 1D CNN layers with kernel sizes of 7, 11, and 15 to explore the information diversity of RNA nucleotides in different local neighborhoods. After we average the output of three 1D CNN layers, a projection head is used to linearly transform the averaging embedding *E* and the final output is calculated as follows:

(4) $$R^{s}=\text{ReLU}(W_{c}^{2}(\text{ReLU}(W_{c}^{1}E))),$$

where $R^{s}\in R^{p\times d}=[r_{1}^{s},r_{2}^{s},\ldots ,r_{p}^{s}]$ is the sequence embedding for RNA nucleotides, $W_{c}^{1}$ and $W_{c}^{2}$ are trainable parameters. The RNA sequence embedding is denoted as $R^{s}\in R^{d}=\text{Mean}(R^{s})=\frac{1}{p}\sum_{i=1}^{p}r_{i}^{s}$.

### 2.3 Small molecule feature extraction module

#### 2.3.1 Small molecule graph information

Extracting features from molecular graphs can effectively mine the chemical information of small molecules. By using the open-source software RDKit, we transform the SMILES of a small molecule into a 2D topology structure, where atoms serve as nodes and bonds serve as edges. The node feature is sourced from the atomic feature description matrix of DeepChem (Ramsundar *et al.* 2019). Then, we apply GCN to learn the graph embedding for the small molecule. The calculation process of *l*th GCN layer is as follows:

(5) $$M^{g,l}=\text{ReLU}\left({\tilde{D}}^{-\frac{1}{2}}\tilde{A}{\tilde{D}}^{-\frac{1}{2}}M^{g,l-1}W_{e}^{l}\right),$$

where $M^{g,l}$ is the atomic hidden representation of small molecule in the *l*th layer, $\tilde{A}$ is the adjacency matrix of small molecule graph with self-connection, $\tilde{D}$ is the degree matrix of $\tilde{A},\;\mathrm{and}\;W_{e}^{l}$ is a trainable parameter. Finally, for a small molecule with *q* atoms, the graph embedding for these *q* atoms is $M^{g}\in R^{q\times d}=[m_{1}^{g},m_{2}^{g},\ldots ,m_{q}^{g}]$. The graph embedding for the small molecule is denoted as $M^{g}\in R^{d}=\text{Mean}(M^{g})=\frac{1}{q}\sum_{i=1}^{q}m_{i}^{g}$.

#### 2.3.2 Small molecule sequence information

SMILES is a text language used to describe the chemical structure of a molecule. To explore the semantic information contained in SMILES, we employ the Transformer (Vaswani *et al.* 2017) encoder to extract pairwise interactions between atomic tokens. Inspired by Du *et al.* (2023) and He *et al.* (2023), we first use an atomic-level SMILES tokenizer to convert SMILES to an input vector. Then, we utilize a Transformer encoder which consists of *L* Transformer blocks. Each Transformer block further consists of two components, a multihead attention layer containing parallel self-attention layers and a feed-forward layer. Taking *M^s^* as the input of a Transformer layer with *u* heads, the calculation process of *i*th head is as follows. Note that we omit the residual connection and the layer normalization after the multiattention layer and the feed-forward layer.

(6) $$\begin{array}{c} {\text{Head}}_{i}=\text{Softmax}\left(\frac{M^{s}W_{i}^{Q}{\left(M^{s}W_{i}^{K}\right)}^{T}}{\sqrt{d}}\right)M^{s}W_{i}^{V} \end{array},$$

where $W_{i}^{Q},\;W_{i}^{K}$, and $W_{i}^{V}$ are trainable parameters. Here, the attention head employs Query ($M^{s}W_{i}^{Q}$), Key ($M^{s}W_{i}^{K}$), and Value ($M^{s}W_{i}^{V}$) matrices to perform scaled dot-product attention, enabling the model to focus on different parts of the input. Then, we concatenate the outputs from *u* heads and obtain MultiHead$\left(M^{s}\right)$ as the output of the multihead attention layer. Taking the *l*th Transformer block as example, the feed-forward layer is calculated as

(7) $$M^{s,l}=\text{ReLU}\left(\text{MultiHead}(M^{s,l-1})\cdot W_{1}^{f,l}+b_{1}^{f,l}\right)W_{1}^{f,l}+b_{1}^{f,l},$$

where $M^{s,l}$ is the output of the *l*th Transformer block, and $W_{1}^{f,l}$ and $W_{2}^{f,l}$ are trainable parameters, $b_{1}^{f,l}$ and $b_{2}^{f,l}$ are bias, $\frac{1}{\sqrt{d}}$ is a scaling factor. After *L* Transformer blocks, the final output of the Transformer encoder $M^{s,L}$ is denoted as $M^{s}\in R^{q\times d}=[m_{1}^{s},m_{2}^{s},\ldots ,m_{q}^{s}]$, which represents the learned sequence embedding for *q* atoms. The sequence embedding for the small molecule is denoted as $M^{s}\in R^{d}=\text{Mean}(M^{s})=\frac{1}{q}\sum_{i=1}^{q}m_{i}^{s}$.

### 2.4 Cross-fusion module

Cross-attention mechanism can be employed to fully fuse RNA and small molecule information. As the Transformer is the most widely used attention-based architecture, we propose a novel Transformer-based cross-fusion module to integrate RNA and small molecule information.

The cross-fusion module includes two inputs, $\left[R^{g},R^{s}\right]$ from RNA in Section 2.2 and $\left[M^{g},M^{s}\right]$ from small molecule in Section 2.3. Each input is composed of the embeddings from both graph and sequence views. To empower the model with the ability to recognize sources of inputs, we introduce a graph-view segment embedding *S^g^* and a sequence-view segment embedding *S^s^* as shown in Equation (8). The segment embedding approach enables the model to discern and assign appropriate weights to the information derived from both views, thereby achieving more effective attention learning for following cross-fusion module.

(8) $$\begin{array}{cc} & R_{\mathrm{input}}^{c}=[R_{\mathrm{input}}^{c,g},R_{\mathrm{input}}^{c,s}]=[R^{g}+S^{g},R^{s}+S^{s}],\\ & M_{\mathrm{input}}^{c}=[M_{\mathrm{input}}^{c,g},M_{\mathrm{input}}^{c,s}]=[M^{g}+S^{g},M^{s}+S^{s}], \end{array}$$

where $R_{\mathrm{input}}^{c}$ and $M_{\mathrm{input}}^{c}$ are the inputs of cross-fusion Transformer.

We design two parallel multicross-attention layers to replace the traditional multiattention layer in the Transformer encoder. Specifically, while traditional multiattention layer can only capture the relationships within the same input, our multicross-attention layers extend this capability by enabling the model to handle inputs from different sources and calculate their relationships. Therefore, the two parallel multicross-attention layers enable the cross-fusion Transformer to consider the fine-grained interaction between RNA and small molecule from their respective views. Taking $R^{c}=[R^{c,g},R^{c,s}]$ and $M^{c}=[M^{c,g},M^{c,s}]$ as the inputs of multicross-attention layers, the cross-attention in *i*th head is calculated as follows, where CrossHead${}_{i}^{R}$ is for RNA view and CrossHead${}_{i}^{M}$ is for small-molecule view as shown in Fig. 1.

(9) $$\begin{array}{cc} & {\text{CrossHead}}_{i}^{R}=\text{Softmax}\left(\frac{R^{c}W_{R,i}^{Q}{\left(M^{c}W_{R,i}^{K}\right)}^{T}}{\sqrt{d}}\right)M^{c}W_{R,i}^{V},\\ & {\text{CrossHead}}_{i}^{M}=\text{Softmax}\left(\frac{M^{c}W_{M,i}^{Q}{\left(R^{c}W_{M,i}^{K}\right)}^{T}}{\sqrt{d}}\right)R^{c}W_{M,i}^{V}, \end{array}$$

where $R^{c}W_{R,i}^{Q}$ and $M^{c}W_{M,i}^{Q}$ are Query matrices, $R^{c}W_{R,i}^{K}$ and $M^{c}W_{M,i}^{K}$ are Key matrices, and $W_{R,i}^{Q},\;W_{R,i}^{K},\;W_{R,i}^{V},\;W_{M,i}^{Q},\;W_{M,i}^{K}$, and $W_{M,i}^{V}$ are trainable parameters. It should be noted that the two inputs of cross-attention heads are ultimately composed of fine-grained embeddings, where *R^c^* is composed of nucleotide embeddings from the graph view and sequence view and *M^c^* is composed of atom embeddings from the graph view and sequence view. Taking the cross-attention of RNA perspective as an example, we first calculate the pairwise attention score between each fine-grained embedding of the RNA query vector $R^{c}W_{R,i}^{Q}$ and small molecule key vector $M^{c}W_{R,i}^{K}$. Ignoring the trainable parameter and Softmax function, the attention score matrix can be represented as $R^{c}{\left(M^{c}\right)}^{T}=\left[\begin{array}{cc} R^{c,g}M^{c,g} & R^{c,g}M^{c,s}\\ R^{c,s}M^{c,g} & R^{c,s}M^{c,s} \end{array}\right]\in R^{2p\times 2q}$, including intraview pair (the embeddings of nucleotide and atom both come from graph-view or sequence-view) and cross-view pair (the embeddings of nucleotide and atom come from graph-view or sequence-view, respectively). Then, we multiply the cross-attention score by the small molecule value vector $M^{c}W_{R,i}^{V}\in R^{2q\times d}$ to achieve atomic information weighting of small molecules on each nucleotide in RNA, allowing the model to adjust the attention of nucleotides embedding at different positions and views based on the information of small molecule.

Finally, similar to Equation (6), we concatenate the outputs of multiple cross-attention heads for both the RNA and small molecule perspectives, and obtain the output of a cross-fusion Transformer through feed-forward layers. After computing through *L* cross-attention Transformer blocks and the mean operation, we can generate the output of cross-fusion module $[R^{c,g},R^{c,s}]\in R^{2\times d}$ and $[M^{c,g},M^{c,s}]\in R^{2\times d}$.

### 2.5 Affinity prediction module

After feature extraction and fusion of the previous three modules, we obtained four RNA embeddings $R^{g},\;R^{s},\;R^{c,g}$, and $R^{c,s}$, and four small molecule embeddings $M^{g},\;M^{s},\;M^{c,g}$, and $M^{c,s}$. First, we obtain RNA embedding R and small molecule embedding M through mean operation, concatenation operation and a two-layered MLP separately. The calculation process is as follows:

(10) $$R=\text{MLP}([\text{Mean}(R^{g},R^{c,g}),\text{Mean}(R^{s},R^{c,s})]),$$

(11) $$M=\text{MLP}([\text{Mean}(M^{g},M^{c,g}),\text{Mean}(M^{s},M^{c,s})]),$$

where the MLP includes dropout operation and ReLU as activate function. Finally, we used a two-layered MLP to fuse RNA embedding $R\in R^{d}$ and small molecule embedding $M\in R^{d}$:

(12) $$\hat{y}=\text{MLP}([R,M]),$$

where $\hat{y}\in R$ is the predicted binding affinity. The model is trained to minimize the mean squared error between predicted and actual binding affinities. During the training process, the learning rate is 5e-4, the batch size is 16, hidden size dimension *d* is 128, the dropout rate is 0.2, and the weight decay is 1e-7. The model is optimized using the Adam optimizer. All the experiments are conducted on three NVIDIA RTX4090 GPUs and are repeated three times with different seeds.

## 3 Results

### 3.1 Datasets and baselines

In order to comprehensively evaluate the model’s ability to predict RNA–small molecule binding affinity, we evaluated DeepRSMA on two datasets in this study, including R-SIM dataset (Krishnan *et al.* 2023) for cross-validation (CV) and an independent dataset. First, we processed the R-SIM dataset and obtained 1439 instances across 341 RNAs and 749 small molecules, including RNA sequences, SMILES strings of small molecules, and their binding affinity values. We applied the negative logarithm of the dissociation constant (pKd) as the metric to measure the binding affinities between RNAs and small molecules. In addition, we collected the binding affinity data of 48 compounds for HIV-1 transcriptase response RNA obtained by surface plasmon resonance from Cai *et al.* (2022) for an independent test.

We employed the following three different experimental setups: (1) *Cross-validation test*: We conducted stratified 5-fold and 10-fold CV, which ensures that each fold maintains the distribution of target values in the dataset, making each fold representative of the overall data distribution. (2) *Blind test*: To evaluate the model’s performance on unseen data, we designed three different dataset splits for blind testing, comprising blind RNA, blind small molecule, and blind both RNA and small molecule. Using blind RNA as an example, we divided the RNA–small molecule pairs into five folds based on RNAs, ensuring that RNAs in each fold did not overlap. Five-fold CV was used to verify the performance of the model under this setting. (3) *Independent test*: Due to the limited amount of data in the independent dataset, we followed the setting in Krishnan *et al.* (2024) and only used the 282 viral RNA data of R-SIM dataset for training, while using the 48 RNA–small molecule pairs related to HIV-1 transcriptase response (trans-activation response—TAR) RNA for testing. To ensure the independence of the experiments, we further filtered out the RNA and small molecules from the training set that were similar to those in the independent test set. After removing 2 RNAs and 10 small molecules from the training data, we obtained an updated training set comprising 141 RNA–small molecule pairs and their affinity scores, while the test set data remained unchanged. More details of the independent test can be found in the Supplementary Material.

To evaluate the performance of DeepRSMA, we adopt three metrics, namely Pearson’s correlation coefficient (PCC), Spearman’s correlation coefficient (SCC), and root mean squared error (RMSE) between predicted and actual binding affinities. The final results were obtained from the average of three repeated experimental results with different seeds. We compared our methods with three categories of methods, comprising nine baselines. The baselines of machine learning method includes support vector machine (SVM) (Hearst *et al.* 1998), k-nearest neighbors (KNN) (Fix and Hodges 1951), and XGBoost (Chen and Guestrin 2016). The deep learning baselines involve GCN, GAT, and Transformer. The last category of baselines consists of three drug-target binding affinity prediction models, including DeepCDA (Abbasi *et al.* 2020), DeepDTAF (Wang *et al.* 2021), and GraphDTA (Nguyen *et al.* 2021). More details of the baseline methods can be found in the Supplementary Material.

### 3.2 Performance comparison under multiple settings

#### 3.2.1 Cross-validation results

The performance comparison between DeepRSMA and other baselines under cross-validation setting is shown in the left half of Table 1. DeepRSMA demonstrates the state-of-the-art performance across all the evaluation metrics under five-fold CV, achieving PCC of 0.784, SCC of 0.786, and RMSE of 0.904. Compared to the second best method in terms of each metric, the corresponding improvement of PCC, SCC, and RMSE are 1.6%, 1.7%, and 2.0%, respectively. Our DeepRSMA achieves the highest PCC and SCC, indicating that the distribution of the predicted binding affinity values can fit the distribution of the actual values very well. Meanwhile, having the lowest RMSE illustrates that DeepRSMA achieve the most accurate predictions among all tested methods. In addition, we also conducted a Student’s *t*-test for our DeepRSMA and GraphDTA (i.e. the second best performer under five-fold CV). The *P*-values for PCC, SCC, and RMSE are all less than 0.05, indicating that the performance improvement achieved by DeepRSMA is statistically significant. Additionally, the results of 10-fold CV can be found in Supplementary Table S1, which also demonstrates the leading performance of DeepRSMA.

**Table 1.** Results under five-fold CV.

Methods	Regression task			Classification task		
	PCC↑	SCC↑	RMSE↓	Specificity↑	BACC↑	AUC↑
SVM	0.706	0.714	0.994	0.535	0.756	0.756
KNN	0.671	0.684	1.038	0.578	0.761	0.760
XGBoost	0.755	0.765	0.922	0.597	0.782	0.781
GCN	0.715	0.717	1.046	0.539	0.757	0.879
GAT	0.715	0.716	1.012	0.526	0.748	0.882
Transformer	0.699	0.695	1.067	0.601	0.783	0.901
DeepCDA	0.746	0.743	0.982	0.604	0.790	0.917
DeepDTAF	0.751	0.747	0.957	0.619	0.795	0.914
GraphDTA	0.772	0.773	0.928	0.611	0.787	0.907
DeepRSMA	**0.784**	**0.786**	**0.904**	**0.650**	**0.807**	**0.920**

Note: The best performance for each metric is marked in bold, while the second-best performance is marked in underlined.

**Figure 2.**
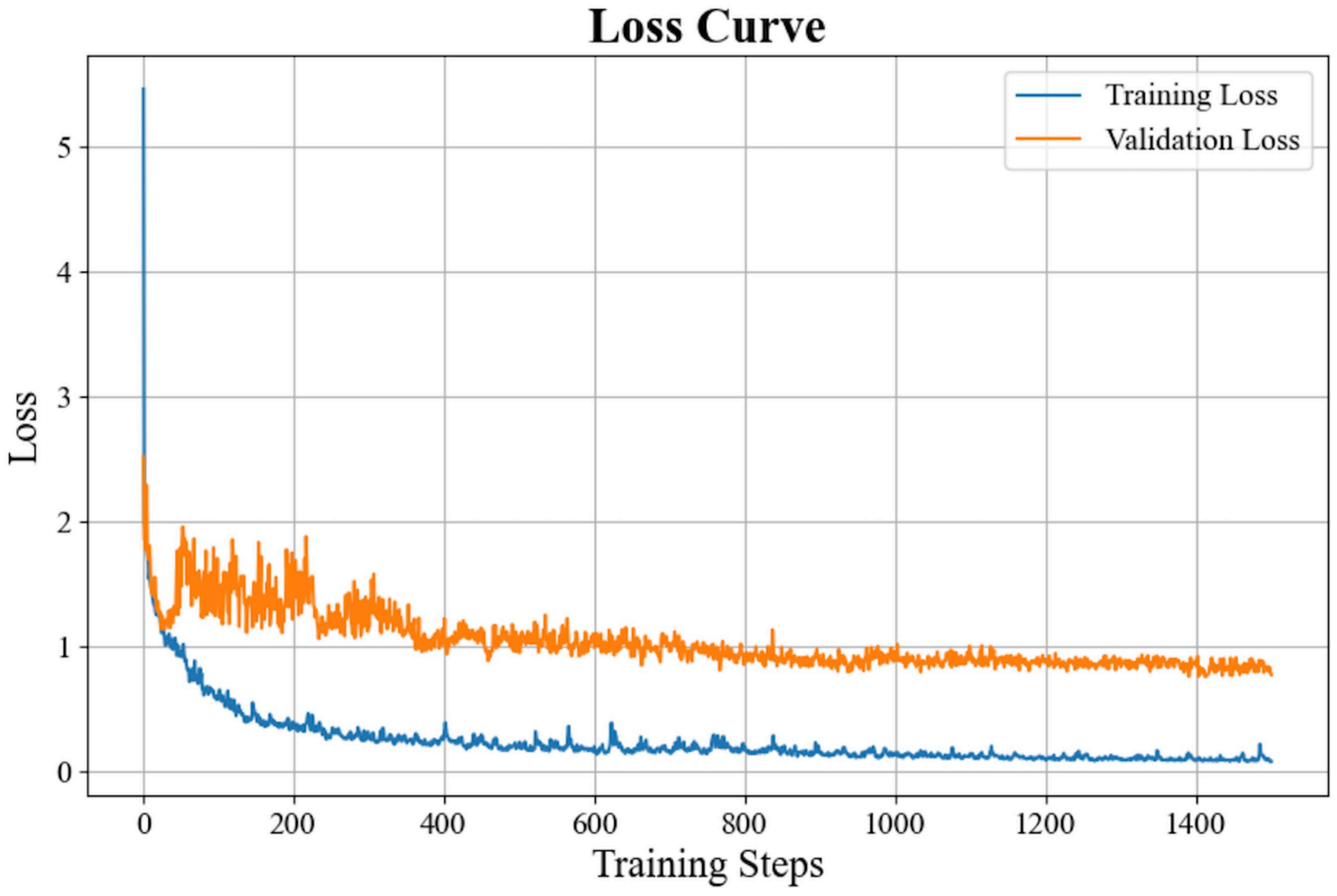
The training and validation curves from one of the experiments in the five-fold CV.

We also plotted the training and validation loss curves during five-fold cross-validation. Figure 2 shows the loss curve from one of the experiments in the five-fold cross-validation. The validation loss closely follows the training loss without significant divergence, suggesting that the model is not overfitting to the training data. This consistency between the curves supports the robustness and generalizability of the model. The complete results for all the five experiments can be found in Supplementary Fig. S4.

To provide a more comprehensive evaluation, we transformed the regression task into a classification task using a threshold value of 4.0 from Yazdani *et al.* (2023). After conducting a statistical analysis, the new classification dataset comprises 1181 positive samples and 258 negative samples, resulting in an imbalanced distribution between the two classes. We conducted five-fold cross-validation and calculated three evaluation metrics, i.e. specificity, balanced accuracy (BACC) score, and the area under the receiver operating characteristic curve (AUC). The results are shown in the right half of Table 1. In terms of these three metrics, DeepRSMA achieves improvements over the second best performers by 5.0%, 1.5%, and 0.3%. The results demonstrate that our model performs well in the classification task. More results can be found in Supplementary Table S2.

#### 3.2.2 Blind test results

In order to evaluate the ability of various models to predict binding affinities for novel RNAs and small molecules, we implemented three blind settings “Blind RNA”, “Blind small molecule,” and “All blind” for blind test. The results of “Blind RNA” and “Blind small molecule” are shown in Table 2. Since the blind test is more challenging than cross-validation, the performance of all the tested models decrease. However, DeepRSMA retains the best performance in all the metrics of both blind settings, achieving average PCC of 0.642, SCC of 0.633, and RMSE of 1.099. Note that Transformer and XGBoost also perform well as they can achieve the second best performance in terms of certain metrics as shown in Table 2. Compared with Transformer, our DeepRSMA can achieve average improvements for PCC, SCC, and RMSE across two blind settings by 12.8%, 11.6%, and 12.5%, respectively. Compared with XGBoost, the average improvements for PCC, SCC, and RMSE across two blind settings are 9.7%, 10.7%, and 2.8%. These results demonstrate DeepRSMA’s robustness when encountering novel RNAs or small molecules. We attribute the performance to the capability of our proposed cross-fusion module to uncover fine-grained feature interaction between RNAs and small molecules. More results of “All blind” can be found in Supplementary Table S3, further demonstrating the superior performance of DeepRSMA.

**Table 2.** Blind test results for various methods.

Methods	Blind RNA			Blind small molecule		
	PCC↑	SCC↑	RMSE↓	PCC↑	SCC↑	RMSE↓
SVM	0.465	0.459	1.233	0.649	0.653	1.069
KNN	0.438	0.433	1.276	0.603	0.609	1.131
XGBoost	0.496	0.463	1.216	0.673	0.680	1.046
GCN	0.429	0.448	1.351	0.642	0.648	1.146
GAT	0.418	0.430	1.357	0.665	0.658	1.092
Transformer	0.563	0.566	1.223	0.575	0.567	1.248
DeepCDA	0.493	0.470	1.263	0.653	0.647	1.136
DeepDTAF	0.560	0.548	1.184	0.651	0.647	1.130
GraphDTA	0.538	0.530	1.289	0.684	0.670	1.118
DeepRSMA	**0.582**	**0.576**	**1.157**	**0.702**	**0.689**	**1.041**

Note: The best performance for each metric is marked in bold, while the second-best performance is marked in underlined.

#### 3.2.3 Independent test results

The independent test is a crucial step for objectively evaluating model’s generalization ability. The results of independent test are shown in Table 3. DeepRSMA achieves PCC of 0.490, SCC of 0.449, and RSME of 0.920, significantly outperforming all the baselines. In terms of PCC, SCC, and RMSE, DeepRSMA achieves improvements over the second best performers by 23.7%, 21.1%, and 5.0%. Due to the inferior generalization capability, the machine learning baselines (e.g. SVM, KNN, and XGBoost) perform poorly in the independent test. Among the deep learning methods, our method’s superior performance can be attributed to the following two reasons. First, the RNA and small molecule feature extraction modules of DeepRSMA can process the features from sequence and graph perspectives to obtain more comprehensive range of information. Second, the cross-fusion module we proposed is capable of effectively integrating the extracted RNA and small molecule features. To summarize, the independent test result demonstrates that DeepRSMA can capture the general patterns from RNAs, small molecules and their feature interactions, and thus has better generalization ability.

**Table 3.** Performance comparison under independent setting.

Methods	PCC↑	SCC↑	RMSE↓
SVM	−0.101	−0.090	1.116
KNN	0.097	−0.012	1.144
XGBoost	−0.169	−0.209	1.383
GCN	0.297	0.409	1.025
GAT	0.258	0.381	1.017
Transformer	0.396	0.412	0.968
DeepCDA	0.305	0.293	1.025
DeepDTAF	0.077	0.052	1.106
GraphDTA	0.301	0.316	1.012
DeepRSMA	**0.490**	**0.499**	**0.920**

Note: The best performance for each metric is marked in bold, while the second-best performance is marked in underlined.

### 3.3 Performance comparison on six RNA subtypes

**Figure 3.**
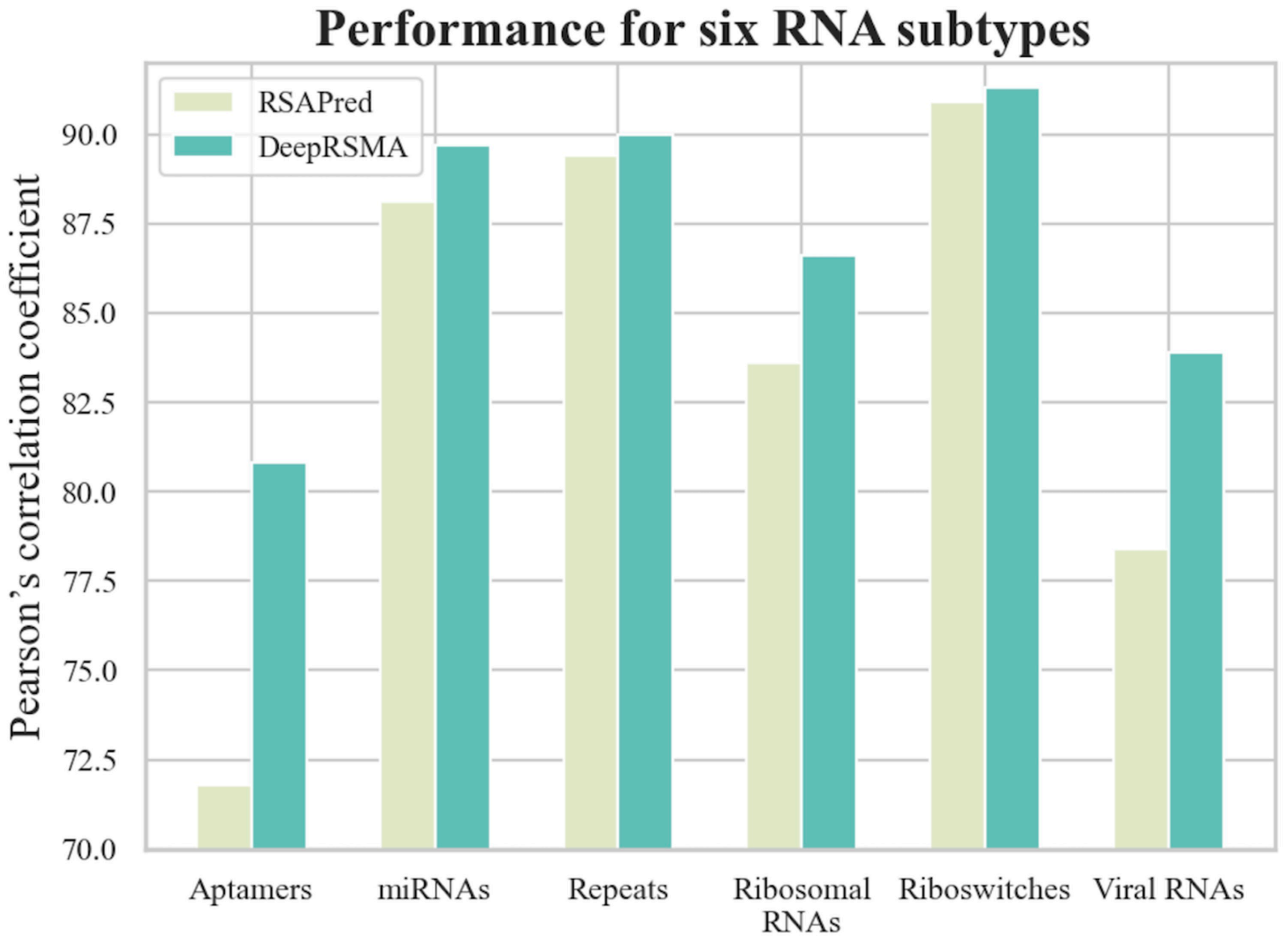
Performance comparison between DeepRSMA and RSAPred on six RNA subtypes.

In order to compare with the state-of-the-art method RSAPred (Krishnan *et al.* 2024), which constructs models based on different RNA subtypes, we evaluated DeepRSMA on the data from six RNA subtypes, including aptamers, miRNAs, repeats, ribosomal RNAs, riboswitches, and viral RNAs. The six RNA subtype datasets are obtained from RSAPred and the performance comparison on stratified 10-fold CV is shown in Fig. 3. Even without specifically selecting features for different RNA subtypes, DeepRSMA outperformes RSAPred on six RNA subtypes, particularly for aptamers and viral RNAs (improving PCC by 12.4% and 6.6%, respectively). However, compared to other subtypes, DeepRSMA does not have a substantial improvement in repeats and riboswitches as shown in Fig. 3. The reason may be that the data of miRNAs and riboswitches is limited, with only 146 and 100 affinity data, respectively. The scarcity of data affects DeepRSMA’s ability to capture the interaction patterns between RNAs and small molecules and hinders the performance improvement. DeepRSMA achieves an average PCC of 0.871 on the six RNA subtypes, which is 4.1% higher than that of RSAPed. The scatter plots for both predicted and actual binding affinities on the six RNA subtypes can be found in Supplementary Fig. S6, demonstrating strong performance of DeepRSMA. These results indicate that DeepRSMA attains the state-of-the-art performance without relying on selecting specific features based on RNA subtypes, suggesting its broader applicability.

### 3.4 Ablation study

To verify the contribution of each component in DeepRSMA, we devised three variants and evaluated their performance using five-fold CV. DeepRSMA without graph view (w/o Gra) removes the component for extracting graph features from RNAs and small molecules. DeepRSMA without sequence view (w/o Seq) removes the component for obtaining sequence features from RNAs and small molecules. DeepRSMA without cross-fusion module (w/o Fusion) removes the cross-fusion module.

The results under five-fold CV are shown in the left half of Table 4. The noticeable decrease in performance for DeepRSMA (w/o Gra) underscores the importance of graph information, which can model the connections between RNA nucleotides and topological structures containing small molecule chemical information. And the performance of DeepRSMA (w/o Seq) illustrates mining the rich biomolecular pattern information contained in the specific arrangement of nucleotides and atoms in sequence can help models predict RNA–small molecule binding affinity more accurately. Lastly, the inferior performance of DeepRSMA (w/o Fusion) after removing the cross-fusion module demonstrates that our proposed cross-fusion module endows DeepRSMA with the ability to learn RNA and small molecule binding patterns at fine-grained scales. To further validate the importance of each component in our model, we conducted an additional ablation study using the independent test setting, which presents a more challenging scenario. The results are shown in the right half of Table 4 and demonstrate that as the difficulty of the task increases, the impact of removing components become more pronounced. It confirms that each component of our model is crucial, particularly under challenging conditions.

**Table 4.** Ablation study of DeepRSMA.

Methods	Five-fold cross-validation test			Independent test		
	PCC↑	SCC↑	RMSE↓	PCC↑	SCC↑	RMSE↓
w/o Gra	0.762	0.759	0.955	0.378	0.316	0.981
w/o Seq	0.769	0.770	0.916	0.421	0.403	0.979
w/o Fusion	0.777	0.774	0.916	0.372	0.331	0.979
DeepRSMA	**0.784**	**0.786**	**0.904**	**0.490**	**0.499**	**0.920**

Note: The best performance for each metric is marked in bold.

### 3.5 Interpretability analysis

**Figure 4.**
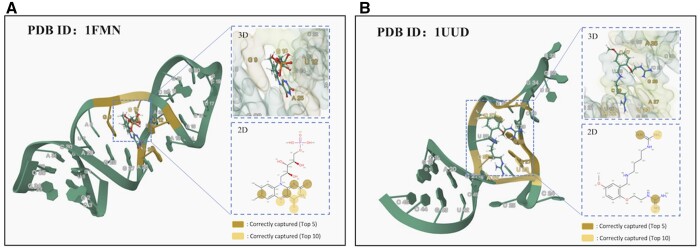
Attention visualization for RNA–small molecule binding affinity. (A) PDB ID: 1FMN. (B) PDB ID: 1UUD. The left part is the solution NMR structure of the complex. The right part is the 2D pose of ligand and the 3D binding pose of ligand with pocket. In the solution structure and 3D binding pose, the important RNA nucleotides with high rankings are highlighted. And the important atoms of small molecule with high rankings are labeled in the 2D ligand pose.

Due to the utilization of the cross-attention mechanism in DeepRSMA for predicting the affinity between RNA and small molecule, the magnitude of attention scores can partially demonstrate whether our method effectively focuses on the crucial binding sites of RNAs and small molecules. Specifically, we obtained the attention score matrices of RNA and small molecule from the cross-fusion Transformer, each containing attention scores from the graph and sequence perspectives. We averaged the scores from different perspectives to derive the final attention scores for RNA and small molecule and then visualized them structurally. Here, we chose 1UUD and 1FMN from the Protein Data Bank (PDB) database. As shown in Fig. 4, the top 5 nucleotides and atoms of attention weights are highlighted in dark yellow, while the top 10 are highlighted in light yellow. The highlighted nucleotides and atoms are crucial binding sites that have been experimentally verified.

1FMN shows a complex of flavin mononucleotide (FMN) bound to a 35-nucleotide RNA aptamer. The isoalloxazine structure of FMN slots into the helical space between the mismatched G9–G27 pair and the G10–U12–A25 base triple. The edge of the isoalloxazine ring, resembling uracil, pairs with A26’s Hoogsteen side through a couple of hydrogen bonds (Fan *et al.* 1996). DeepRSMA successfully assigns high attention scores to four binding sites, including G9, G10, U12, and A26. Specifically, all the nucleotides, except for G10, are predicted to be within the top 5 positions. It can be observed that our model has identified most of the nucleotides in the binding pocket. Among the top 10 small molecule atoms recognized by our model, 8 of them are located within the isoalloxazine ring.

1UUD is an NMR structure that shows the complex of the bis-guanidine compound rbt203 and TAR element (RNA) from HIV-1. In this case, seven RNA binding sites, including A22, U23, A27, G28, A35, C37, and C39, are correctly captured. Among them, A22 and U23 are located in the major groove region of the RNA. The guanidinium groups of the small molecule may form cation–*π* stacking interactions with them, which facilitate the binding of the small molecule to the RNA (Davis *et al.* 2004). Our model can successfully identify the two guanidine groups of rbt203 as key interacting groups. The visualization results demonstrate that DeepRSMA is capable of capturing the binding sites on RNAs and the substructures of the small molecules involved in the interaction and has the potential to provide insights for RNA-targeted drug discovery.

### 3.6 Case study on SMN2 pre-mRNA

To validate DeepRSMA’s ability to predict RNA–small molecule affinity with reasonable accuracy, we applied DeepRSMA to predict the binding affinity of SMN2 pre-mRNA and two small molecules with therapeutic potential for SMA. SMA is a genetic neuromuscular disorder caused by mutations in the SMN1 gene, leading to the loss of motor neurons and progressive muscle weakness (Talbot and Tizzano 2017). The therapeutic approach for SMA involves targeting the SMN2 gene’s pre-mRNA splicing process to increase the production of a stable form of the SMN protein. Risdiplam and branaplam are two of the most promising drugs for the treatment of SMA. Risdiplam (Ratni *et al.* 2018) is the first orally available small molecule approved by the FDA for the treatment of SMA, enhancing the production of functional SMN protein, and represents a significant advancement in the treatment of this genetic disorder. Branaplam (Cheung *et al.* 2018), developed as an SMN2 splicing modifier, has undergone clinical trials. We note that these two drugs are not in the R-SIM dataset. Moreover, we further calculated their Tanimoto coefficients (Du *et al.* 2023) with the small molecules in the R-SIM dataset. The highest and average Tanimoto coefficients between risdiplam and small molecules in R-SIM are 0.266 and 0.091, respectively. These two scores for branaplam are 0.453 and 0.090. Their low Tanimoto coefficient scores indicate that the two drugs are not similar to the small molecules in the R-SIM dataset (Maggiora *et al.* 2014). More descriptions for these two drugs can be found in the Supplementary Material. The binding affinity values predicted by DeepRSMA for risdiplam and branaplam with SMN2 pre-mRNA are 4.98 and 4.75, respectively, which are very close to the experimentally obtained values of 4.92 and 4.74 from titration experiments (Malard *et al.* 2024). The results of our case study demonstrate that DeepRSMA has high predictive ability for RNA–small molecule binding affinities.

## 4 Conclusion

In this work, we introduce a novel deep learning method to predict RNA–small molecule binding affinity, namely DeepRSMA. DeepRSMA extracts fine-grained information of RNA and small molecule from sequence view and graph view. Moreover, a cross-fusion module is designed to learn fine-grained interactions between different views of RNA and small molecule. DeepRSMA outperforms all tested baselines under numerous experimental settings. The results of interpretability experiments illustrate that our cross-fusion module can capture key regions of RNA–small molecule binding which may aid structure-based drug design. These results show that DeepRSMA has the potential to accelerate the discovery of RNA-targeted drugs.

## Supplementary Material

btae678_Supplementary_Data

## Data Availability

The codes and datasets are available online at https://github.com/Hhhzj-7/DeepRSMA.

## References

[btae678-B1] Abbasi K , Razzaghi P, Poso A et al DeepCDA: deep cross-domain compound–protein affinity prediction through LSTM and convolutional neural networks. Bioinformatics 2020;36:4633–42. 32462178 10.1093/bioinformatics/btaa544

[btae678-B3] Cai Z , Zafferani M, Akande OM et al Quantitative structure–activity relationship (QSAR) study predicts small-molecule binding to RNA structure. J Med Chem 2022;65:7262–77. 35522972 10.1021/acs.jmedchem.2c00254 PMC9150105

[btae678-B4] Caprara MG , Nilsen TW. RNA: versatility in form and function. Nat Struct Biol 2000;7:831–3. 11017186 10.1038/82816

[btae678-B5] Chen J , Hu Z, Sun S et al Interpretable RNA foundation model from unannotated data for highly accurate RNA structure and function predictions. arXiv, arXiv:2204.00300, 2022, preprint: not peer reviewed.

[btae678-B6] Chen T , Guestrin C. XGBoost: a scalable tree boosting system. In: *Proceedings of the 22nd ACM SIGKDD International Conference on Knowledge Discovery and Data Mining, KDD ’16*. pp. 785–94. New York, NY, USA: ACM, 2016.

[btae678-B7] Cheung AK, Hurley B, Kerrigan R *et al*. Discovery of small molecule splicing modulators of survival motor neuron-2 (SMN2) for the treatment of spinal muscular atrophy (SMA). *J Med Chem* 2018;61:11021–36. 10.1021/acs.jmedchem.8b01291 30407821

[btae678-B8] Childs-Disney JL , Yang X, Gibaut QMR et al Targeting RNA structures with small molecules. Nat Rev Drug Discov 2022;21:736–62. 35941229 10.1038/s41573-022-00521-4 PMC9360655

[btae678-B9] Costales MG , Childs-Disney JL, Haniff HS et al How we think about targeting RNA with small molecules. J Med Chem 2020;63:8880–900. 32212706 10.1021/acs.jmedchem.9b01927 PMC7486258

[btae678-B10] Davis B , Afshar M, Varani G et al Rational design of inhibitors of HIV-1 TAR RNA through the stabilisation of electrostatic “hot spots”. J Mol Biol 2004;336:343–56. 14757049 10.1016/j.jmb.2003.12.046

[btae678-B11] Du B-X , Long Y, Li X et al CMMS-GCL: cross-modality metabolic stability prediction with graph contrastive learning. Bioinformatics 2023;39:btad503. 37572298 10.1093/bioinformatics/btad503 PMC10457661

[btae678-B12] Fan P , Suri AK, Fiala R et al Molecular recognition in the FMN–RNA aptamer complex. J Mol Biol 1996;258:480–500. 8642604 10.1006/jmbi.1996.0263

[btae678-B13] Feng Y , Huang S-Y. ITScore-NL: an iterative knowledge-based scoring function for nucleic acid–ligand interactions. J Chem Inf Model 2020;60:6698–708. 33291885 10.1021/acs.jcim.0c00974

[btae678-B7107148] Fix E, , Hodges JL. Discriminatory Analysis: Nonparametric Discrimination, Consistency Properties. Randolph Field, Texas: USAF School of Aviation Medicine, 1951.

[btae678-B14] Grimberg H , Tiwari VS, Tam B et al Machine learning approaches to optimize small-molecule inhibitors for RNA targeting. J Cheminform 2022;14:4. 35109921 10.1186/s13321-022-00583-x PMC8811966

[btae678-B15] Guilbert C , James TL. Docking to RNA via root-mean-square-deviation-driven energy minimization with flexible ligands and flexible targets. J Chem Inf Model 2008;48:1257–68. 18510306 10.1021/ci8000327 PMC2910576

[btae678-B16] He H , Chen G, Chen CY-C et al NHGNN-DTA: a node-adaptive hybrid graph neural network for interpretable drug–target binding affinity prediction. Bioinformatics 2023;39:btad355. 37252835 10.1093/bioinformatics/btad355 PMC10287904

[btae678-B17] Hearst MA , Dumais ST, Osuna E et al Support vector machines. IEEE Intell Syst their Appl 1998;13:18–28.

[btae678-B18] Kairys V , Baranauskiene L, Kazlauskiene M et al Binding affinity in drug design: experimental and computational techniques. Expert Opin Drug Discov 2019;14:755–68. 31146609 10.1080/17460441.2019.1623202

[btae678-B19] Kipf TN, Welling M. Semi-supervised classification with graph convolutional networks. In: 5th International Conference on Learning Representations (ICLR), 2017.

[btae678-B20] Krishnan SR et al R-SIM: a database of binding affinities for RNA-small molecule interactions. J Mol Biol 2023;435:167914. 36495921 10.1016/j.jmb.2022.167914

[btae678-B21] Krishnan SR , Roy A, Gromiha MM et al Reliable method for predicting the binding affinity of RNA-small molecule interactions using machine learning. Brief Bioinform 2024;25:bbae002. 38261341 10.1093/bib/bbae002 PMC10805179

[btae678-B22] Krizhevsky A , Sutskever I, Hinton GE et al Imagenet classification with deep convolutional neural networks. Commun ACM 2017;60:84–90.

[btae678-B23] Maggiora G , Vogt M, Stumpfe D et al Molecular similarity in medicinal chemistry: miniperspective. J Med Chem 2014;57:3186–204. 24151987 10.1021/jm401411z

[btae678-B24] Malard F , Wolter AC, Marquevielle J et al The diversity of splicing modifiers acting on a-1 bulged 5-splice sites reveals rules for rational drug design. Nucleic Acids Res 2024;52:4124–36. 38554107 10.1093/nar/gkae201 PMC11077090

[btae678-B25] Nguyen T , Le H, Quinn TP et al GraphDTA: predicting drug–target binding affinity with graph neural networks. Bioinformatics 2021;37:1140–7. 33119053 10.1093/bioinformatics/btaa921

[btae678-B26] O’Keefe L. FDA approves oral treatment for spinal muscular atrophy. FDA News Release 2020.

[btae678-B27] Ramsundar B, Eastman P, Walters P et al *Deep Learning for the Life Sciences: Applying Deep Learning to Genomics, Microscopy, Drug Discovery, and More*. O’Reilly Media, Inc., 2019.

[btae678-B28] Ratni H, Ebeling M, Baird J *et al*. Discovery of risdiplam, a selective survival of motor neuron-2 (SMN2) gene splicing modifier for the treatment of spinal muscular atrophy (SMA). *J Med Chem* 2018;61:6501–17. 10.1021/acs.jmedchem.8b00741 30044619

[btae678-B29] Singh J , Paliwal K, Litfin T et al Predicting RNA distance-based contact maps by integrated deep learning on physics-inferred secondary structure and evolutionary-derived mutational coupling. Bioinformatics 2022;38:3900–10. 35751593 10.1093/bioinformatics/btac421 PMC9364379

[btae678-B30] Szulc NA , Mackiewicz Z, Bujnicki JM et al Structural interaction fingerprints and machine learning for predicting and explaining binding of small molecule ligands to RNA. Brief Bioinform 2023;24:bbad187. 37204195 10.1093/bib/bbad187

[btae678-B31] Talbot K , Tizzano E. The clinical landscape for SMA in a new therapeutic era. Gene Ther 2017;24:529–33. 28644430 10.1038/gt.2017.52 PMC5628264

[btae678-B32] Vaswani A, Shazeer N, Parmar N et al Attention is all you need. Adv Neural Inf Process Syst 2017;30.

[btae678-B33] Veličković P, Cucurull P, Casanova A et al Graph attention networks. arXiv, arXiv:1710.10903, 2017, preprint: not peer reviewed.

[btae678-B34] Wang K , Zhou R, Li Y et al DeepDTAF: a deep learning method to predict protein–ligand binding affinity. Brief Bioinform 2021;22:bbab072. 33834190 10.1093/bib/bbab072

[btae678-B35] Wang K , Zhou R, Wu Y et al Rlbind: a deep learning method to predict RNA–ligand binding sites. Brief Bioinform 2023;24:bbac486. 36398911 10.1093/bib/bbac486

[btae678-B36] Warner KD , Hajdin CE, Weeks KM et al Principles for targeting RNA with drug-like small molecules. Nat Rev Drug Discov 2018;17:547–58. 29977051 10.1038/nrd.2018.93 PMC6420209

[btae678-B37] Yazdani K et al Machine learning informs RNA-binding chemical space. Angew Chem 2023;135:e202211358. 10.1002/anie.202211358 PMC9992102 36584293

[btae678-B38] Yu A-M , Choi YH, Tu M-J et al RNA drugs and RNA targets for small molecules: principles, progress, and challenges. Pharmacol Rev 2020;72:862–98. 32929000 10.1124/pr.120.019554 PMC7495341

