# Supplementary material for: DeepRSMA: a cross-fusion-based deep learning method for RNA–small molecule binding affinity prediction
Source: Bioinformatics. 2024 Nov 14;40(12):btae678. doi: 10.1093/bioinformatics/btae678 (PMC11646567; doi:10.1093/bioinformatics/btae678)
Supplement: btae678_Supplementary_Data [file btae678_supplementary_data.pdf]

DeepRSMA: a cross-fusion based deep learning method for RNA-small molecule binding affinity prediction

Zhijian Huang [1], Yucheng Wang [2], Song Chen [1], Yaw Sing Tan [3], Lei Deng [1,*] and Min Wu [2,*]

*To whom correspondence should be addressed.

[1]School of Computer Science and Engineering, Central South University, Changsha, 410083, China and
[2]Institute for Infocomm Research, Agency for Science, Technology and Research (A*STAR), 138632, Singapore and
[3]Bioinformatics Institute, Agency for Science, Technology and Research (A*STAR), 138671, Singapore.

Contents

1 Details of baseline methods **2**

2 Details of features and parameters in DeepRSMA **3**

3 Details of independent test and abation study **5**

4 More results under cross-validation setting **7**

5 Visualization of training loss with training steps **8**

6 More results on classification task **9**

7 More results on blind setting **12**

8 More results on six RNA subtypes **13**

1 Details of baseline methods

To evaluate the performance of DeepRSMA, we compare our methods with nine baselines: support vector machine (SVM) [1], random forest (RF) [2], XGBoost [3], graph convolutional network [4], graph attention network [5], Transformer, DeepCDA [6], DeepDTAF [7] and GraphDTA [8].

The input feature of machine learning baselines SVM, RF and XGBoost are the concatenation of molecular Morgan fingerprints [9] and RNA nucleotide index vectors. Both GCN and GAT are algorithms used to learn complex relationships between nodes in graph structured data. The graph structure data for the inputs of these two baselines is the same as DeepRSMA. The Transformer is a deep learning method widely used in Natural Language Processing (NLP). The input for the transformer handling RNA is the initial nucleotide embedding, while the input for the transformer handling small molecule is the atomic-level embedding. For these three classic deep learning methods, we finally concatenated the embeddings of RNA and small molecule, and achieved affinity prediction through a 2-layered MLP. In order to compare with other methods for predicting binding affinity, we choose some general and adaptable methods, including DeepCDA, DeepDTAF and GraphDTA. DeepCDA is a method for predicting compound-protein affinity, which uses CNN and long short-term memory networks (LSTM) to learn sequence features of compounds and proteins, and proposes a two side attention mechanism to fuse their descriptors. DeepDTAF is a deep learning method based on CNN that can capture local and global contextual features to predict protein–ligand binding affinity. GraphDTA represents drugs as graph and uses different graph neural networks to predict drug-target affinity.

2 Details of features and parameters in DeepRSMA

DeepRSMA takes three RNA features and two small molecule features as input. The RNA features include an RNA contact map, a nucleotide one-hot encoding and the pre-trained RNA embedding from RNA-FM. The small molecule features include a molecular structure that contains atom features and edge features, and an atomic-level SMILES tokenizer embedding. After feature extraction and cross-fusion module, we can obtain 4 RNA embeddings and 4 small molecule embeddings, which is used as input for the affinity prediction module. Taking RNA as an example, the four RNA features are $\mathcal{R}^g \in \mathbb{R}^d$, $\mathcal{R}^s \in \mathbb{R}^d$, $\mathcal{R}^{c,g} \in \mathbb{R}^d$ and $\mathcal{R}^{c,s} \in \mathbb{R}^d$, where d is the hidden size dimension. $\mathcal{R}^g$ and $\mathcal{R}^s$ refer to the RNA graph embedding and sequence embedding from the feature extraction module, respectively. $\mathcal{R}^{c,g}$ and $\mathcal{R}^{c,s}$ represent the cross-view graph embedding and sequence embedding of RNA, respectively, obtained after the computation of cross attention with small molecule embeddings in the cross-fusion module. Finally, we obtain the final RNA embedding $\mathcal{R} \in \mathbb{R}^d$ and small molecule $\mathcal{M} \in \mathbb{R}^d$ embedding through the following equations:

$$\mathcal{R} = \mathrm{MLP}([\mathrm{Mean}(\mathcal{R}^g, \mathcal{R}^{c,g}), \mathrm{Mean}(\mathcal{R}^s, \mathcal{R}^{c,s})]), \quad (1)$$

$$\mathcal{M} = \mathrm{MLP}([\mathrm{Mean}(\mathcal{M}^g, \mathcal{M}^{c,g}), \mathrm{Mean}(\mathcal{M}^s, \mathcal{M}^{c,s})]), \quad (2)$$

where the MLP inculdes dropout operation and ReLU as activate function. We also provide additional details regarding the selection of the hidden size dimension d. Fig. S1 shows the computational costs and performance when the hidden size dimension d takes different values of 32, 64, 128, and 256. In particular, we use RMSE as performance indicator and the number of parameters as the indicator of computational costs.

As shown in Fig. S1, it is evident that when the dimension d increased from 128 to 256, the number of model parameters increased significantly, while the performance improvement was minimal. Specifically, when the hidden size dimension d increased from 64 to 128, the model parameters increased by 83.9%, while performance also improved by 2.2%. However, when the hidden size increased from 128 to 256, the model parameters increased by 163.7%, but performance only improved by 1.3%. Additionally, a larger hidden size also leads to a higher risk of overfitting. Therefore, we selected a hidden size of 128 and the total number of parameters in the model is approximately 3.55M.

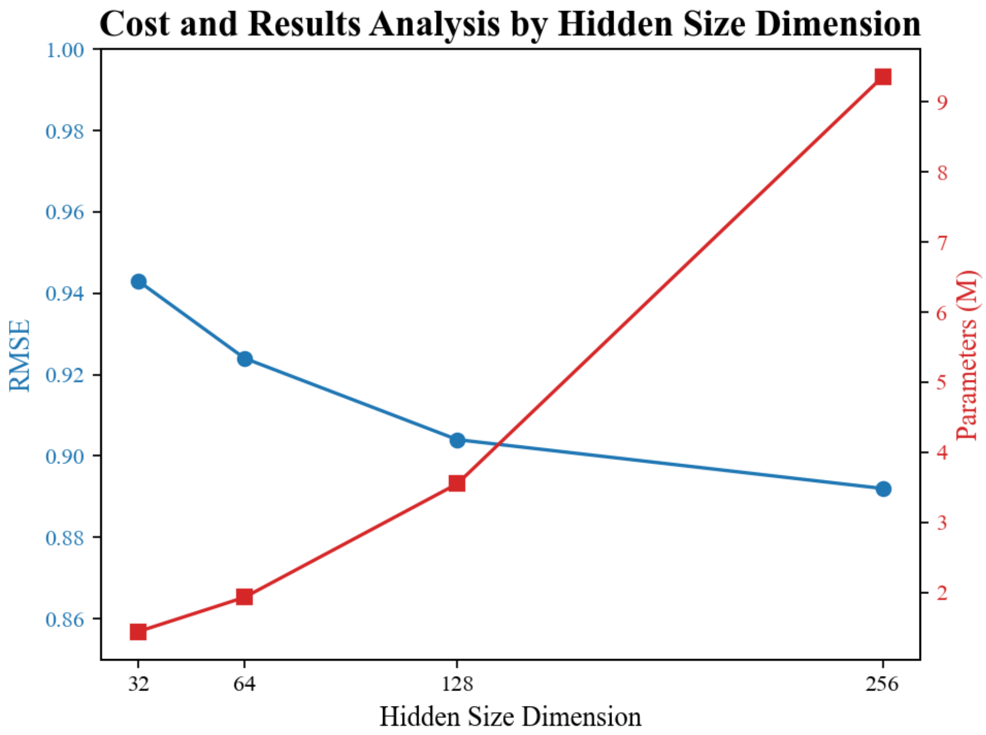

Figure S1: Variation of cost (number of parameters) and results (RMSE) with different hidden size dimensions, where the x-axis represents hidden size dimension, the left y-axis indicates cost, and the right y-axis represents results.

3 Details of independent test and abation study

We followed (Krishnan et al., 2024) to collect the data for independent test. Specifically, the training set includes 282 viral RNAs extracted from the R-SIM dataset (Krishnan et al., 2023), while the test set comprises 48 RNA-small molecule pairs related to HIV-1 transcriptase response (TAR) RNA data from (Cai et al., 2022). We confirm that there are no common RNA-small molecule pairs between the training and test sets. However, it is possible that there are repeated or similar RNAs or small molecules across two sets. To ensure the independence of the test data, we further processed the training data as follows.

First, we identified that HIV-1 TAR RNA was present in the training data and removed it. We also utilized CD-HIT-EST (Li and Godzik, 2006) to cluster the RNA nucleotide sequences. HIV-1 TAR RNA and HIV-2 TAR RNA were grouped into the same cluster, and thus we removed HIV-2 TAR RNA from the training set as well.

Second, we used ECFP4 fingerprints for small molecules and then calculated their Tanimoto coefficients (TC). In particular, two small molecules are considered to be highly similar if their TC value is greater than 0.85 (Maggiora et al, 2014). By calculating TC values between small molecules in the training and test data as shown in Fig. S2, we identified 10 small molecules in the training set that were highly similar to those in the test set. These 10 small molecules were subsequently removed from the training data.

In summary, we removed 2 RNAs and 10 small molecules from the training data, resulting in an updated training set comprising 141 RNA-small molecule pairs and their affinity scores. Meanwhile, the test data was unchanged. We conducted the independent test using this new training data. The data of training and test comes from different sources. As shown in Fig. S2, there is a noticeable difference in their data distributions. This difference indicates that the independent test presents a more challenging task compared to the other two test settings.

For Risdiplam and Branaplam, we calculated their Tanimoto Coefficients (TC) with the small molecules in R-SIM dataset. The highest and average TC scores between Risdiplam and small molecules in R-SIM are 0.266 and 0.091, respectively. These two scores for Branaplam are 0.453 and 0.090. These low TC scores indicate that the two molecules are dissimilar to those in R-SIM dataset [10]. Therefore, the two small molecules, Risdiplam and Branaplam, are independent from the training data.

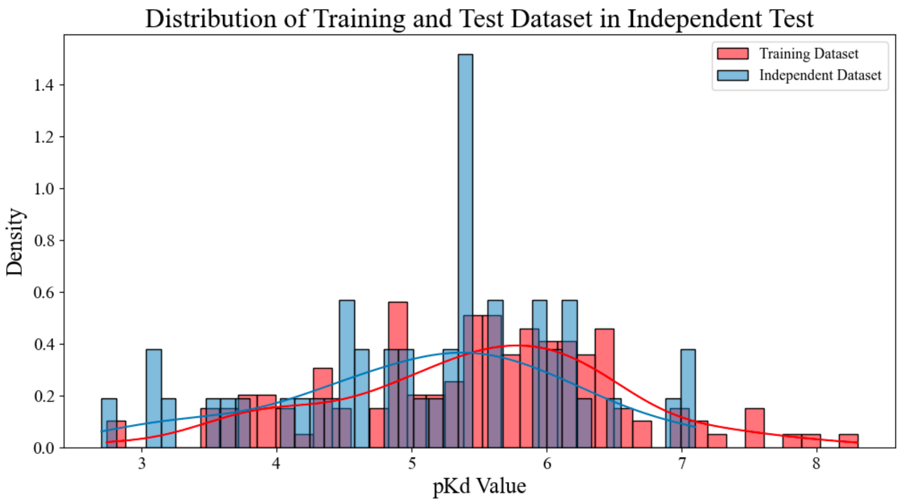

Figure S2: The histogram of pKd value for the training and test set in independent test with an overlaid kernel density estimate curve, illustrating the distribution of the data and probability density.

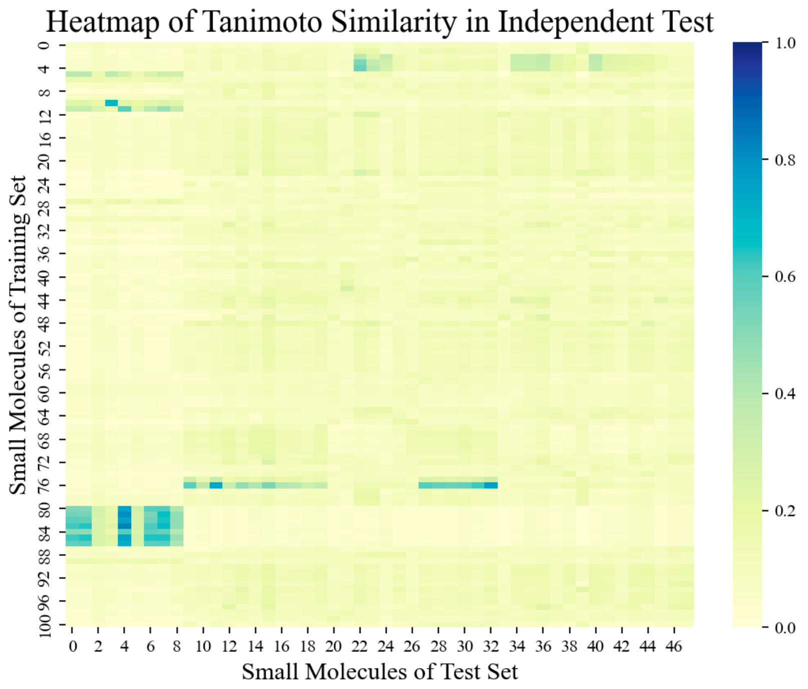

Figure S3: The Tanimoto Similarity in independent test. The y-axis represents the small molecules from training set, and the x-axis represents the small molecules from test set.

4 More results under cross-validation setting

The results of 10-fold CV are shown in Table S1. DeepRSMA demonstrates the state-of-art method across all evaluation metrics in 10-fold CV, achieving PCC of 0.806, SCC of 0.806 and RMSE of 0.862 on 10-fold CV. The corresponding relative improvement of PCC, SCC and RMSE are 1.6%, 2.5% and 3.6%. To verify the reliability of the results of cross-validation setting, we conduct the student's t-test for our DeepRSMA and other baseline methods. All the P-values are less than 0.05 in both 5-fold CV and 10-fold CV, except for the P-value with D in RMSE during 10-fold cross-validation, where it is $6.716e^{-2}$. The reason is the significant variability in the RMSE values of DeepDTAF across three repeated experiments under 10-fold cross-validation, which are 1.040, 0.924, and 0.919, respectively. These results indicate that DeepRSMA achieve state-of-the-art performance under cross-validation setting.

Table S1: Performance comparison on 10-fold CV.

Methods	PCC↑	SCC↑	RMSE↓
SVM	0.720	0.726	0.975
KNN	0.687	0.699	1.016
XGBoost	0.765	0.770	0.906
GCN	0.733	0.728	1.006
GAT	0.749	0.745	0.956
Transformer	0.730	0.729	0.997
DeepCDA	0.769	0.770	0.949
DeepDTAF	0.772	0.768	0.961
GraphDTA	0.793	0.786	0.894
DeepRSMA	**0.806**	**0.806**	**0.862**

Note: The best performance for each metric is marked in bold, while the second-best performance is marked in underlined.

5 Visualization of training loss with training steps

We plotted the loss function during training and also included the validation loss curve for each fold in the five-fold cross-validation process. These plots provide a comprehensive view of the model's performance and confirm that it is not overfitting.

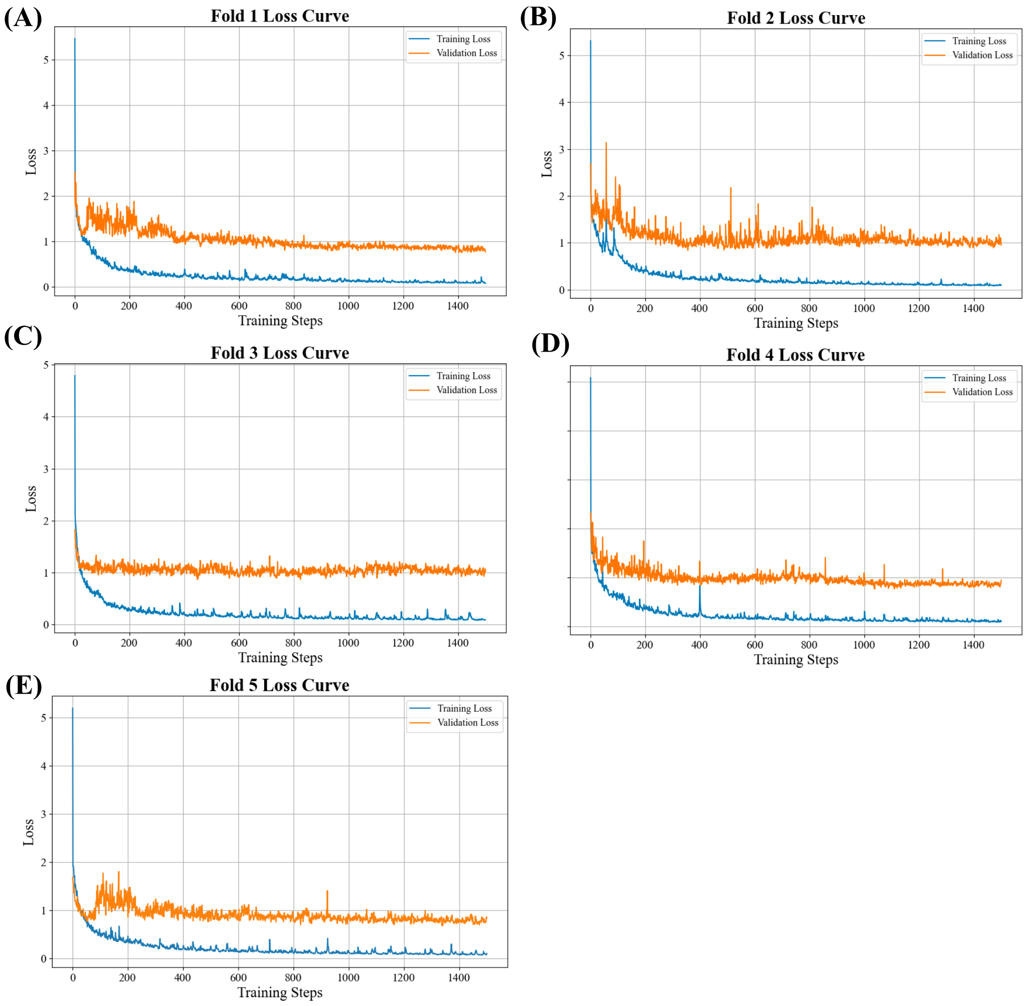

Figure S4: Training and validation loss curves during 5-fold cross-validation.

6 More results on classification task

Relying solely on PCC (and SCC, RMSE) may not capture all aspects of the model's performance, especially in cases where the distribution of data points could influence these metrics.

To address this problem, we have conducted an additional experiment by transforming the regression task into a classification task using a threshold value of 4.0 from Yazdani et al (2023). A pKd value above 4 indicates that the RNA-small molecule pair is a positive sample with high affinity, while a low value indicates a negative sample with lower affinity. After conducting a statistical analysis, the new classification dataset comprises 1,181 positive samples and 258 negative samples, resulting in an imbalanced distribution between the two classes. The data distribution is shown in Figure 1.

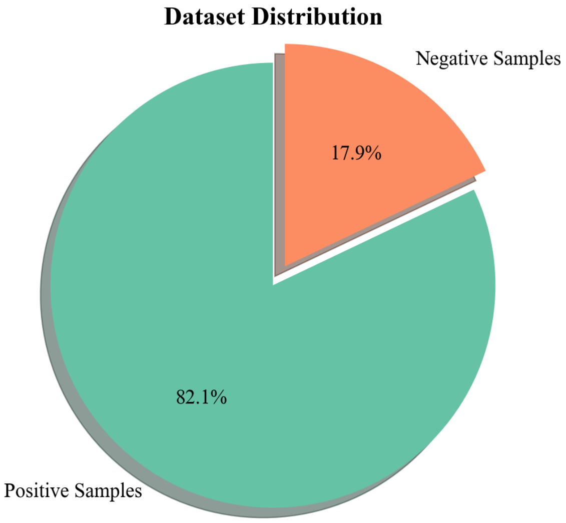

Figure S5: Pie chart illustrating the distribution of positive and negative samples in the classification task.

We conducted 5-fold cross-validation and calculated precision, recall, the area under the receiver operating characteristic curve (AUC), specificity and balanced accuracy (BACC) scores. The definitions of these metrics are as follows:

$$\text{Precision} = \frac{TP}{TP + FP} \tag{3}$$

$$\text{Recall} = \frac{TP}{TP + FN} \tag{4}$$

$$\text{Specificity} = \frac{TN}{TN + FP} \tag{5}$$

$$\text{BACC} = \frac{1}{2}\left(\frac{TP}{TP + FN} + \frac{TN}{TN + FP}\right) \tag{6}$$

where TP denotes true positives, FP denotes false positives, TN denotes true negatives, and FN denotes false negatives. AUC is calculated by integrating the area under the ROC curve, which plots the True Positive Rate against the False Positive Rate across different classification thresholds. The results are as follows:

Table S2: Classification performance comparison on 5-fold CV.

Methods	TP	Precision	Recall	Specificity	BACC	AUC
SVM	1154	0.906	**0.977**	0.535	0.756	0.756
KNN	1114	0.911	0.943	0.578	0.760	0.761
XGBoost	1141	0.917	0.966	0.597	0.782	0.781
GCN	1153	0.907	<u>0.976</u>	0.539	0.757	0.879
GAT	1146	0.904	0.971	0.526	0.748	0.882
Transformer	1140	0.918	0.965	0.601	0.783	0.901
DeepCDA	1151	0.920	0.975	0.604	0.790	<u>0.917</u>
DeepDTAF	1148	<u>0.922</u>	0.972	<u>0.619</u>	<u>0.795</u>	0.914
GraphDTA	1138	0.920	0.963	0.611	0.787	0.907
DeepRSMA	1139	**0.927**	0.964	**0.650**	**0.807**	**0.920**

Note: The best performance for each metric is marked in bold, while the second-best performance is marked in underlined.

To provide a more comprehensive evaluation of model performance,we also assess how well these methods predict negative pairs. For this purpose, we report three additional metrics: Specificity, Balanced Accuracy (BACC), and the Area Under the ROC Curve (AUC). Here, specificity in Eq. (5) shows the fraction of negative pairs that are correctly predicted. BACC is the average of recall and specificity. AUC score measures the trade-off between the true positive rate (i.e., recall) and false positive rate (i.e., 1-specificity) across all possible classification thresholds. Given that BACC and AUC consider accuracy for both classes, they are particularly suitable for imbalanced datasets. In terms of these three metrics, DeepRSMA achieves improvements over the second best performers by 0.3%, 5.0% and 1.5%. The results demonstrate that our model performs well in the classification task.

7 More results on blind setting

Compared to “Blind RNA” and “Blind small molecule”, "All Blind" is a more difficult scenario. The performance comparison results under "All Blind" setting are shown in Table S3. Compared to the next best method GraphDTA in cross-validation test, the improvement of PCC, SCC and RMSE are 19.4%, 19.0% and 26.9%, which the improvement is obviously much higher compared to the improvement observed in both “Blind RNA” and “Blind small molecule”. These results demonstrate robustness and performance superiority of DeepRSMA.

Table S3: Performance comparison under "All blind" setting.

Methods	PCC↑	SCC↑	RMSE↓
SVM	0.210	0.314	<u>1.284</u>
KNN	0.209	0.211	1.331
XGBoost	0.223	0.177	1.351
GCN	0.335	0.336	1.411
GAT	0.360	0.382	1.518
Transformer	<u>0.498</u>	0.444	1.696
DeepCDA	0.372	0.365	1.372
DeepDTAF	0.458	<u>0.448</u>	1.417
GraphDTA	0.480	0.447	1.677
DeepRSMA	**0.573**	**0.532**	**1.229**

Note: The best performance for each metric is marked in bold, while the second-best performance is marked in underlined.

8 More results on six RNA subtypes

Figure S1 shows the scatter plots of six RNA subtypes. The histograms displayed at the margins depict the comprehensive distribution of true value and predicted value. As illustrated, the data points exhibit a propensity for symmetry around the line y = x in all RNA subtypes, which demonstrates the high accuracy of DeepRSMA in predicting RNA-small molecule affinity for six RNA subtypes.

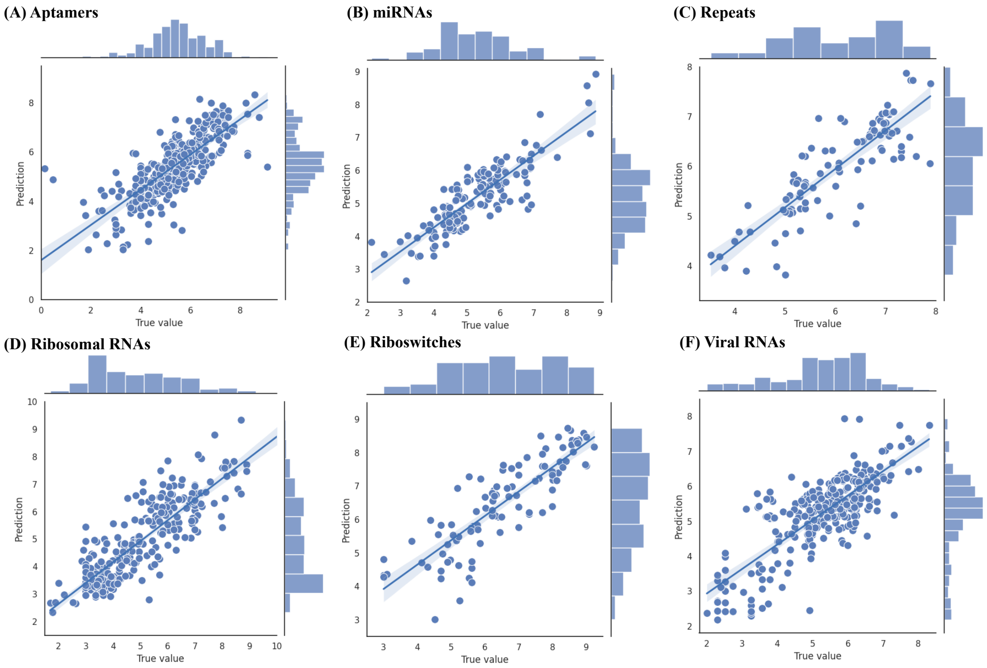

Figure S6: The true affinity against the predicted value on six RNA subtypes. X-axis reflects true values and Y-axis reflects predicted values.

References

[1] Marti A. Hearst, Susan T Dumais, Edgar Osuna, John Platt, and Bernhard Scholkopf. Support vector machines. *IEEE Intelligent Systems and their applications*, 13(4):18–28, 1998.

[2] Leo Breiman. Random forests. *Machine learning*, 45(1):5–32, 2001.

[3] Tianqi Chen and Carlos Guestrin. Xgboost: A scalable tree boosting system. In *Proceedings of the 22nd acm sigkdd international conference on knowledge discovery and data mining*, pages 785–794, 2016.

[4] Thomas N Kipf and Max Welling. Semi-supervised classification with graph convolutional networks. *arXiv preprint arXiv:1609.02907*, 2016.

[5] Petar Veličković, Guillem Cucurull, Arantxa Casanova, Adriana Romero, Pietro Lio, and Yoshua Bengio. Graph attention networks. *arXiv preprint arXiv:1710.10903*, 2017.

[6] Karim Abbasi, Parvin Razzaghi, Antti Poso, Massoud Amanlou, Jahan B Ghasemi, and Ali Masoudi-Nejad. Deepcda: deep cross-domain compound–protein affinity prediction through lstm and convolutional neural networks. *Bioinformatics*, 36(17):4633–4642, 2020.

[7] Kaili Wang, Renyi Zhou, Yaohang Li, and Min Li. Deepdtaf: a deep learning method to predict protein–ligand binding affinity. *Briefings in Bioinformatics*, 22(5):bbab072, 2021.

[8] Thin Nguyen, Hang Le, Thomas P Quinn, Tri Nguyen, Thuc Duy Le, and Svetha Venkatesh. Graphdta: predicting drug–target binding affinity with graph neural networks. *Bioinformatics*, 37(8):1140–1147, 2021.

[9] Harry L Morgan. The generation of a unique machine description for chemical structures-a technique developed at chemical abstracts service. *Journal of chemical documentation*, 5(2):107–113, 1965.

[10] Gerald Maggiora, Martin Vogt, Dagmar Stumpfe, and Jurgen Bajorath. Molecular similarity in medicinal chemistry: miniperspective. *Journal of medicinal chemistry*, 57(8):3186–3204, 2014.
